# Long Term Outcome after Application of the Angio-Seal Vascular Closure Device in Minipigs

**DOI:** 10.1371/journal.pone.0163878

**Published:** 2016-09-28

**Authors:** Lisa Kabelitz, Andrea Nonn, Kay W. Nolte, Omid Nikoubashman, Ahmed Othman, Sarah Heringer, Martin Kramer, Martin Wiesmann, Marc A. Brockmann

**Affiliations:** 1 Department of Diagnostic and Interventional Neuroradiology, RWTH Aachen University Hospital, Aachen, Germany; 2 Institute of Neuropathology, RWTH Aachen University Hospital, Aachen, Germany; 3 Department of Radiology, University Hospital Tübingen, Tübingen, Germany; 4 Department of Veterinary Clinical Sciences, Small Animal Clinic, Justus-Liebig-University, Giessen, Germany; 5 Department of Neuroradiology, University Medical Center of the Johannes Gutenberg University Mainz, Mainz, Germany; Ehime University Graduate School of Medicine, JAPAN

## Abstract

Minipigs are frequently used in (neuro-)interventional research. Longitudinal experiments may require repeated vessel access via the femoral artery. Anticoagulation and incompliance of the animals necessitates the use of a vascular closure device (VCD). The effects of the Angio-Seal VCD in minipigs were longitudinally assessed. Minipig (42±8.4 kg body weight) femoral arteries were sealed using the 8F (n = 6) or 6F (n = 7) Angio-Seal VCD. The pre-interventional femoral artery diameter was 5.1±0.4 mm (4.3–5.8 mm). Sealed puncture sites were analysed angiographically as well as by computed tomography angiography (CTA) for a mean period of 14.1±8.0 weeks (1–22 weeks). All animals were constantly treated with acetylsalicylic acid (ASS) (450 mg/d (n = 7) or 100 mg/d (n = 1)) and clopidogrel (75 mg/d (n = 8)). Non-instrumented (n = 2) and arteries sealed using the VCD (n = 2) were examined histologically. No postoperative hemorrhagic complications were observed. Three arteries were occluded after VCD placement (1 animal diagnosed after 4 weeks (8F), 2 animals after 1 week (6F)) and remained so until the end of the experiments after 22, 12 and 4 weeks, respectively. In one artery a 50% stenosis 8 weeks after application of a 6F Angio-Seal was detected. In 69.2% (n = 9) the VCD was applied without complications. Histopathological analysis of the sealed arterial segments showed subtotal obliteration of the vessel lumen, formation of collagenous tissue and partial damage of the internal elastic lamina. The Angio-Seal VCD prevents relevant hemorrhagic complications in minipigs treated with dual platelet inhibition, but is associated with increased vessel occlusion rates.

## Introduction

Evaluation of novel (neuro-)interventional devices and treatment modalities often necessitates longitudinal experiments with animal models. The pig is an increasingly used animal model [1,2] also in the field of interventional radiology because of its favourable size and comparable peripheral arterial diameter [3]. Additionally, there are some similarities between the pig’s blood coagulation system and the human one [4–7].

If domestic pigs, like German Landrace, are used, their continuous weight gain might pose a problem relating to comparability of the experimental results besides the handling of heavyweight animals [1]. For example at the age of 250 days domestic pigs weigh about 120 kg compared to minipigs with lass than 40 kg. To avoid these problems, minipigs are increasingly being used due to their stable size and stable vessel diameter [8].

Longitudinal endovascular experiments may require repeated vessel access via the femoral artery. Pigs would not accept lengthy immobilisation without anesthesia or applying of a pressure bandage. This missing compliance as well as anticoagulation implies the necessity of a suitable and efficient VCD. Manual compression of the puncture site, especially if pigs received anticoagulation therapy, is not thought to be effective.

Since their introduction in the 1990s different types of VCDs were developed. A frequently used device is the Angio-Seal vascular closure device (St. Jude Medical, St. Paul, MN, USA) [9–11]. Angio-Seal works by compression of the puncture site in a “sandwich technique” in combination with induction of hemostasis by a collagenous sponge [12]. The system consists of three completely biodegradable components: an anchor (made from polylactic and polyglycolic acids) deployed intraarterially, a small extravascular positioned bovine collagenous sponge and a suture of polyglycolic acid, which connects the elements [13,14]. Angio-Seal is available in sizes of 6F and 8F. All components are completely absorbed within 90 days after application [15,16]. Possible adverse events described in humans are hematoma, AV fistula, pseudoaneurysm, late bleeding requiring transfusion, vessel occlusion and stensosis, allergic reaction, foreign body reaction, inflammation and edema [12–14,17–19].

There are no long term experiences regarding the application of VCDs in minipigs. Tellez et al. [20] investigated the absorption of Angio-Seal only sonographically in large domestic Yorkshire pigs. Isfort et al. [21] tested the StarClose (Abott Vascular, Santa Clara, CA, USA) in 20 German Landrace pigs over a period of 28 days. Hofmann et al. [22] examined the applicability of the Sutura SuperStitch (Sutura, Fountain Vally, CA, USA) and Perclose (Abott Vascular, Santa Clara, CA, USA) system in 8 domestic pigs over 4 weeks.

In the present study the outcome after vessel closure using the Angio-Seal VCD was assessed in minipigs up to 22 weeks. Treatment effects were analysed angiographically as well as by CTA and histologically.

## Materials and Methods

### Animals

The experiments were carried out after receiving approval of the governmental animal care office (Landesamt für Natur, Umwelt und Verbraucherschutz Nordrhein-Westfalen, Recklinghausen, Germany). National guidelines for animal ethics, welfare and experimental conduct were followed. 8 female minipigs (Ellegard Göttingen Minipigs A/S, Dalmose, Danmark) (42±8.4 kg initial body weight) were housed under controlled environmental conditions (20°C±1°C, 12:12 h light/dark cycle). The acclimatization period before starting the experiments was 2 weeks. Apart from fasting directly before the experiments all animals received feed and water ad libitum.

### Experimental design

The animals were deployed in various interventional experiments regardless of the present study.

The animals received premedication with azaperone (Stresnil 40 mg ad. us. vet.; Sanochemia Pharmazeutika AG, Neufeld, Austria), atropin (Atropinsulfat, B.Braun Melsungen AG, Melsungen, Germany) and ketamine (10% Ketavet ad us. vet., Zoetis Deutschland GmbH, Berlin, Germany) followed by intubation. The animals were mechanically ventilated with an oxygen-air mixture. Anesthesia was maintained with propofol (Propofol 2% MCT Fresenius; Fresenius Kabi Deutschland GmbH, Bad Homburg, Germany). For analgesia fentanyl (Fentanly-Janssen 0,5 mg, Janssen-Cilag GmbH, Neuss, Germany) was continuously administered. During the treatments the animals were fixed supine.

**Fig 1:**
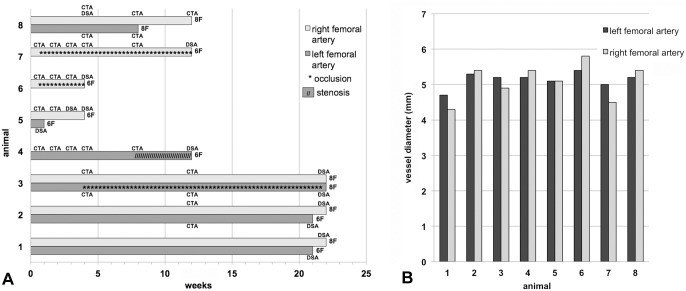
Experimental design. (A) Overview of the number of animals, observation periods, imaging modality, and the condition of the femoral artery after application of an Angio-Seal VCD. (B) Porcine baseline femoral artery diameter.

In the context of these experiments the animals were punctured at the femoral artery and a 6F or 8F sheath was inserted. The puncture site was closed by a 6F (n = 7) or 8F (n = 6) Angio-Seal VCD (Fig 1A). Application of the VCD was performed by an experienced neurointerventionalist.

Starting the day of the intervention all animals were constantly treated with ASS (Aspirin 450 mg/d (n = 7) or 100 mg/d (n = 1); Bayer Vital GmbH, Leverkusen, Germany) and clopidogrel (Iscover, 75 mg/d (n = 8); Orifarm GmbH, Leverkusen, Germany). Three of the animals were treated with 450 mg clopidogrel 24h prior treated. During the intervention animals were treated with ASS (500 mg) and heparin (3000 IU, Heparin-Natrium-5000; ratiopharm GmbH, Ulm, Germany) intravenously to prevent thrombotic events. After the intervention heparin was not received any longer. Using the Multiplate- Analyzer (Dynabyte Medical, München, Germany) the platelet function of all minipigs was verified. Decreased measured values of the ASPI- and ADP-test compared to the initial values indicated effective anticoagulation.

### Follow up imaging

To evaluate the treatment effects after application of the Angio-Seal VCD, follow up imaging of the femoral arteries was performed by CTA (Somatom AS; Siemens Healthcare, Erlangen, Germany) after 1 (n = 4), 2 (n = 4), 3 (n = 3), 4 (n = 6), 8 (n = 4) and 12 (n = 5) weeks (Fig 1A). The minipigs were anesthetized and fixed supine in the described manner. The tube parameters were set to 100 ml contrast agent (Solutrast 300, 300 mg iodine/ml; Altana Pharma AG, Konstanz, Germany) and 30 ml sodium chloride injection with 4 ml / sec. flow.

Treatment effects were also evaluated angiographically (Axiom; Siemens Healthcare) via contralateral vessel puncture access after 1 (n = 1), 3 (n = 1), 4 (n = 3), 12 (n = 2), 21 (n = 2) and 22 (n = 4) weeks. One artery was examined twice. The pigs were anesthetized and fixed supine in the described manner.

The outcome after application of the Angio-Seal VCD was evaluated retrospectively. For that reason, times and modalities of examination differ between some of the animals.

The data were evaluated for stenosis or vessel occlusion. In all cases the initial pre-interventional femoral artery diameter was measured angiographically (Fig 1B).

After the last follow up imaging the animals were sacrificed by intravenous injection of 0.5–1 ml/kg body weight natrium-pentobarbital (Narcoren 16 g/100ml; Merial GmbH, Hallbergmoos, Germany). The treated (n = 13) and untreated (n = 3) femoral arteries were prepared and fixed with 4% formalin.

### Histopathological analyses

The arteries of two animals euthanized at 4 (animal 6) and 12 (animal 7) weeks after instrumentation were analysed histologically. Femoral artery segments harbouring the punctured and sealed vessel region were collected as well as the contralateral non-instrumented arteries. After immersion fixation with 4% buffered formalin the arteries were sectioned at 0.4 cm intervals. Artery sections were embedded in paraffin, cut at 5 μm thickness onto slides and stained with hematoxylin and eosin, kongored, turnbull blue or van Gieson stain. Images of stained slides were captured with a Zeiss Axio Scope.A1 microscope and AxioCam MRc camera (Carl Zeiss Microscopy, Jena, Germany).

## Results

Data evaluation yielded an average pre-interventional femoral artery diameter of 5.1±0.4 mm (Fig 1B). The minipigs were observed for a mean period of 14.1±8.0 weeks (Fig 1A). All treated animals tolerated the procedure well, no postoperative haemorrhagic complications were observed. Technical success rate of the Angio-Seal VCD was 100%.

**Fig 2:**
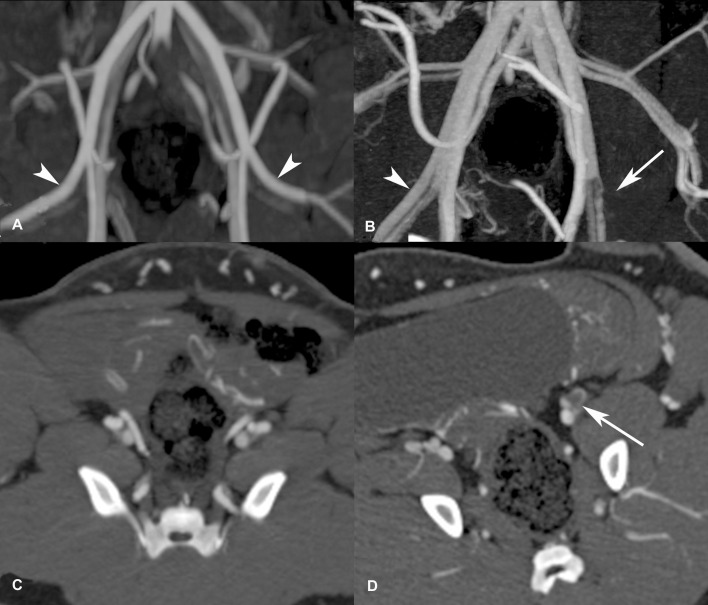
Occluded left femoral artery after application of an 8F Angio-Seal vascular closure device. (A, C) CT-angiography demonstrates the intact femoral arteries prior to arterial puncture (arrow heads in A). (B, D) Four weeks after implantation of an 8F Angio-Seal VCD CT-angiography shows occlusion of the left femoral artery of animal 3 (white arrow in B and D). The animal presented in this figure is one of 3 animals that developed vessel occlusion after application of the Angio-Seal VCD.

After application of the Angio-Seal VCD three arteries were found to be occluded (1 animal after 4 weeks (8F), 2 animals after 1 week (6F)) and remained occluded until the experiments were finished after 22, 12 and 4 weeks, respectively (Figs 1 and 2). As none of the animals showed any impairment, such as pain or lameness, the experiments were continued as scheduled.

**Fig 3:**
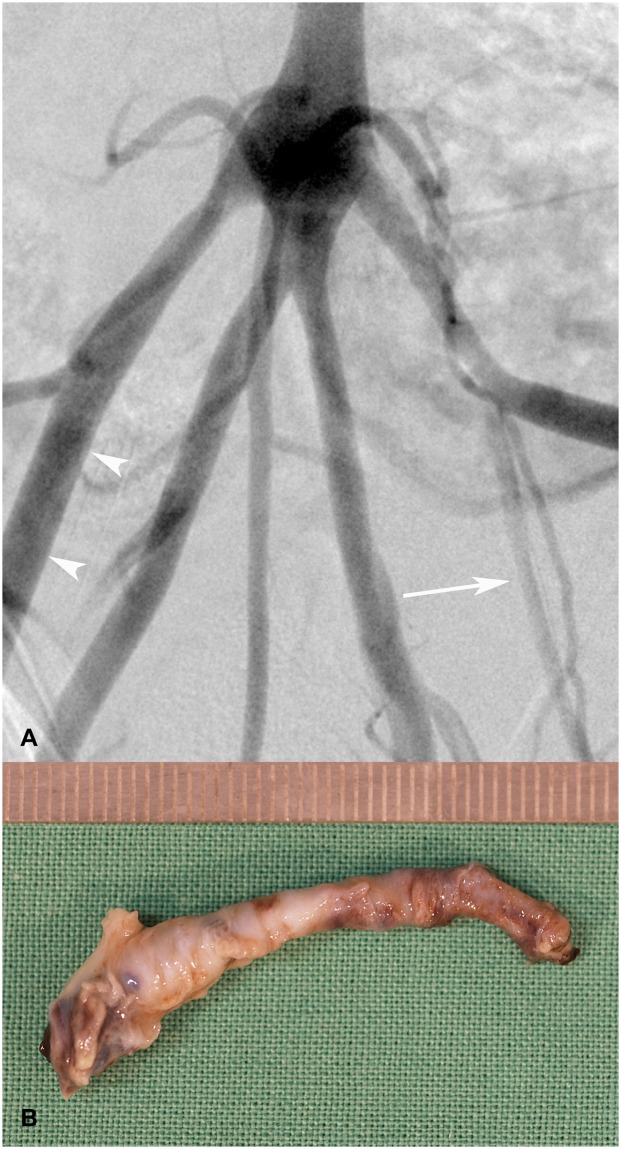
Stenosis of the left femoral artery 12 weeks after application of a 6F Angio-Seal. (A) Digital subtraction angiography shows a local stenosis at the puncture site (white arrow). The normal contralateral artery is shown for comparison (white arrow heads). (B) Macroscopic photography of the left femoral artery shows the pathologically thickened vessel segment at the position of the applied Angio-Seal VCD.

8 weeks after application of a 6F Angio-Seal a 50% stenosis was detected in one artery (animal 4) by CTA and after 12 weeks by digital subtraction angiography (DSA) (Figs 1 and 3).

None of the animals showed signs of distal ischemia or any other impairments.

In the remaining 69.2% (n = 9) Angio-Seal was effectively applied without any complications.

**Fig 4:**
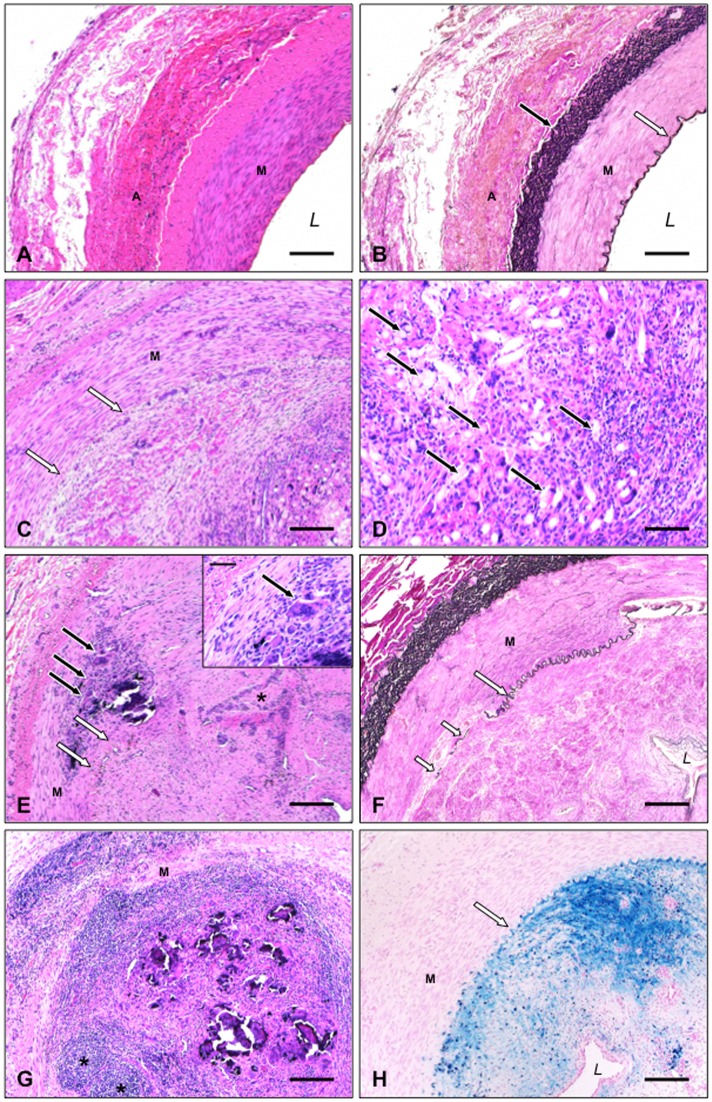
Histopathological findings. (A, B) Segment of normal porcine femoral artery; A adventitia (Tunica externa), M media (Tunica media), L vessel lumen, black arrow external elastic membrane, white arrow internal elastic lamina (A H&E, B van Gieson stain; scale bars = 180 μm). (C) Femoral artery *4 weeks after intervention*. Obliteration of vessel lumen; M media, arrows internal elastic lamina (H&E; scale bar = 180 μm). (D) Multinucleated histiocytes, lymphocytes and scattered eosinophils around copolymer material (arrows), same animal as in C (H&E; scale bar = 90 μm). (E) Advanced connective tissue formation in former intravasal space *12 weeks after endovascular instrumentation*. Focal media calcification surrounded by lymphocytes and multinucleated histiocytes (black arrows). Area with prominent neovascularisation as indicated by asterisk; white arrows internal elastic lamina (H&E; scale bar = 180 μm). Inset: Multinucleated giant cell (arrow) in partially destroyed media (H&E; scale bar = 65 μm). (F) Subtotal obliteration of femoral artery (same animal as in E). Segmental destruction of internal elastic lamina (arrow) with remnants of elastic material (short arrows); (van Gieson stain; scale bar = 180 μm). (G) Prominent inflammatory response (same animal as in E and F). Follicle-like aggregation of small lymphocytes (asterisks) and calcifications embedded in a lympho-histiocytic infiltrate (H&E; scale bar = 180 μm). (H) Considerable hemosiderin deposits (blue staining) at the former vessel lumen (same animal as in E–G); L residual artery lumen (turnbull blue; scale bar = 180 μm).

While cross sections of arteries from the non-instrumented side showed normal arterial vessel architecture without fibrosis, calcification or inflammatory changes (Fig 4A and 4B) sections of sealed arterial segments showed an over 90% to subtotal obliteration of the vessel lumen, mainly due to mesenchymal and foreign body reaction with an excessive formation of collagenous tissue involving the intima and focally extending to the media. Multinucleated histiocytes, lymphocytes and scattered eosinophils around copolymer material also indicate a florid foreign body reaction within the endovascular area. Here and there the internal elastic lamina (IEL) was destroyed with remnants of elastic material lying in fibrotic tissue adjacent to the media (Fig 4F). Hemosiderin deposits indicate hemorrhage within tissue mass at the former vessel lumen.

In the case of the pig finalized 4 weeks (animal 6) after Angio-Seal implantation aggregates of approximately 15 μm in diameter measuring pale eosinophilic corpuscules were discernible in the former vessel lumen. This foreign material obviously representing remnants of the VCD’s intraluminal component turned out to be partially kongophilic and birefringent and was surrounded by granulation tissue dominated by histiocytic cells, several of them being multinucleated (Fig 4C and 4D). Scattered lymphocytes and eosinophilic granulocytes as part of a florid foreign body reaction could also be seen.

12 weeks after instrumentation the foreign material was almost completely degraded and only patchy foci of histiocytes including multinucleated giant cells could be detected, mostly in association with dystrophic calcifications (Fig 4E). Within the soft tissue rich in collagen fibers newly formed capillaries and small vessels occurred (Fig 4E). Extensive hemosiderin deposits within the lumen obliterating fibrous tissue referred to former microbleeding during tissue maturation and neovascularisation (Fig 4H). The affected femoral artery (animal 7) showed pronounced inflammatory changes with an eccentric transmural cellular infiltration of large parts of the vessel wall, focally reaching the adventitia (Fig 4G). This infiltrate was dominated by small reactive lymphocytes which were occasionally assembled in follicular structures, and again histiocytes, granulocytes being only rarely admixed. There were no hints to arterial wall dissection or arteriovenous fistulas in the arterial specimen of any animal.

## Discussion

Since their introduction in the 1990s VCDs are used increasingly with the Angio-Seal being one of the more frequently used VCDs [9–11]. Technically, an anchor is located intraarterially, and a bovine collagenous plug is pressed against the anchor from the outside of the arterial wall. The functionality of the Angio-Seal responses predominantly on practice of the “sandwich technique” supplemented by the thrombogenic properties of the collagen [14,19].

VCDs are of relevance not only for treatment of humans, but also for vessel occlusion of laboratory animals, especially in long term experiments with repeated vessel access and anticoagulant therapy. The pig is one of the species most frequently used for endovascular experimental studies. For long term experiments minipigs should be preferred, because of their stable vessel diameters, stress resistance and suitable size [23,24]. Manual compression of a puncture site, applying of a pressure bandage and lengthy immobilisation are not suitable to occlude puncture sites in pigs without the risk of hemorrhagic complications [21], particularly in the context of anticoagulant or anti-platelet therapy. This implies the necessity of a fast, safe and efficient method of sealing the puncture site.

Hitherto, the use of VCDs has only been investigated in domestic pigs, like German Landrace [20–22]. Detailed examinations of VCDs in minipigs and their long term outcome, however, are missing. The aim of the present study was thus to examine the long term outcome after application of the Angio-Seal VCD in minipigs receiving anticoagulant therapy.

Results of our study suggest that in minipigs the components of the Angio-Seal principally are degradable in accordance with the manufacturer’s data. Thus in 69.2% of cases, regardless of the size of the VCD (6F or 8F) and despite dual platelet inhibition applying ASS and clopidogrel, the application and resorption of the Angio-Seal was successful without any complications. Nevertheless, three animals developed vessel occlusion after application of the Angio-Seal VCD (8F (n = 1) and 6F (n = 2)) (Fig 1A). Furthermore, one artery showed a 50% stenosis 8 weeks after application of a 6F Angio-Seal (Fig 1A). We thus considered the reasons which may have led to these complications.

The initial vessel diameters, measured angiographically, of all femoral arteries were greater than 4 mm (Fig 1B). Thus, the conditions of the manufacturer were complied [14]. Prior to placement of the Angio-Seal, correct positioning of the catheter sheath was verified by contrast injection.

Histologic examinations figured out that despite degradation of the VCD’s components in the animals with subsequent vessel occlusion at the puncture site (i.e. animal 6 and 7 in Fig 1A) mostly intravascular tissue reaction with subtotal luminal obliterative fibrosis occurred (Fig 4A–4H). Whereas Tellez et al. [20] did not report on such complications, several studies in which a polyglycolic and polylactic acid polymer was used described similar reactions after implantation of this material in pigs [25,26]. Allergic reactions due to polyglycolic and polylactic acid are known in humans [14]. It thus can not be ruled out that comparable allergic reactions may also occur in pigs.

The complication rate of approximately 30% may be ascribed to the small sample size of this study. Compared to studies of human medicine with several hundred patients, where complications after application of the Angio-Seal occur less frequently, in the present study a total of 13 arteries were analyzed in 8 pigs. Thus, data from a larger sample size might have altered the reported complication rate.

One of the limitations of the underlying study is that follow-up imaging was performed at heterogenous time points. The reason for this was that the animals were deployed in various interventional experiments regardless of the present study. The follow-up intervals were thus determined by these experiments. Nevertheless, synchronization of imaging intervals would not have relevantly affected the outcome of the study.

Another debatable limitation is the lack of a control group (i.e. manual compression of the femoral artery to achieve bleeding control). In pigs, bleeding control would have been difficult to establish due to the pigs’ anatomy and due to the fact, that the animals are not compliant regarding immobilisation or bedrest.

Finally, only a relatively small number of pigs was examined, which also might have affected the reported complication rate of around 30%.

In conclusion, Angio-Seal effectively prevents relevant bleeding complications in minipigs treated with dual platelet inhibition, hereby allowing longitudinal endovascular studies. It should, however, be noted that rates of vessel occlusion or stenosis might be increased in the context of using this VCD.

## Supporting Information

S1 Fig Physiologically perfused femoral arteries of animal 2 (22 weeks after application of a 6F and 8F Angio-Seal VCD shown by DSA). (TIF)

S2 Fig Physiologically perfused femoral arteries of animal 2 (12 and 22 weeks after application of a 6F and 8F Angio-Seal VCD shown by CTA and DSA). (TIF)

S3 Fig Occluded left femoral artery of animal 3 (4, 12 and 22 weeks after application of a 8F Angio-Seal VCD shown by CTA and DSA). (TIF)

S4 Fig Follow-up imaging using CTA and DSA demonstrated a stenosis of the left femoral artery of animal 4 after application of a 6F Angio-Seal VCD. (TIF)

S5 Fig Physiologically perfused femoral arteries of animal 5 (1, 2, 3 and 4 weeks after application of a 6F Angio-Seal VCD shown by CTA and DSA). (TIF)

S6 Fig Occluded right femoral artery of animal 6 (1, 2, 3 and 4 weeks after application of a 6F Angio-Seal VCD shown by CTA and DSA). (TIF)

S7 Fig Occluded right femoral artery of animal 7 (1, 2, 3, 4, 8 and 12 weeks after application of a 6F Angio-Seal VCD shown by CTA and DSA). (TIF)

S8 Fig Physiologically perfused femoral arteries of animal 8 (4,8 and 12 weeks after application of a 8F Angio-Seal VCD shown by DSA and CTA). (TIF)

## References

[1] Swindle MM, Smith AC. Best practices for performing experimental surgery in swine. J Invest Surg. 2013; 26: 63–71. 10.3109/08941939.2012.693149 23281597

[2] Helke KL, Swindle MM. Animal models of toxicology testing: the role of pigs. Expert Opin Drug Metab Toxicol. 2013; 9: 127–39. 10.1517/17425255.2013.739607 23216131

[3] Ettrup KS, Glud AN, Orlowski D, Fitting LM, Meier K, Soerensen JC, et al Basic surgical techniques in the Gottingen minipig: intubation, bladder catheterization, femoral vessel catheterization, and transcardial perfusion. J Vis Exp. 2011 10.3791/2652 21730947 PMC3197034

[4] Roussi J, André P, Samama M, Pignaud G, Bonneau M, Laporte A, et al Platelet functions and haemostasis parameters in pigs: absence of side effects of a procedure of general anaesthesia. Thromb Res. 1996; 81: 297–305. 10.1016/0049-3848(96)00001-1 8928087

[5] Brophy C, Ito R, Quist W, Rosenblatt MS, Contreras M, Tsoukas A, et al A new canine model for evaluating blood prosthetic arterial graft interactions. J Biomed Mater Res. 1991; 25: 1031–8. 10.1002/jbm.820250809 1833406

[6] Osterman FA, Bell WR, Montali RJ, Novak GR, White RI. Natural history of autologous blood clot embolization in swine. Invest Radiol. 1975; 11: 267–76. 10.1097/00004424-197607000-00003 134002

[7] Siller-Matula JM, Plasenzotti R, Spiel A, Quehenberger P, Jilma B. Interspecies differences in coagulation profile. Thromb Haemost. 2008; 100: 397–404. 10.1160/TH08-02-0103 18766254

[8] Hiebl B, Müller C, Jung F, Hüningen H, Hamm B, Plendl J, et al Macro- and micromorphometric studies of the vascular structures from the Göttingen minipig. Appl Cardiopulm Pathophysiol. 2009; 13: 318–21.

[9] Gargiulo NJ, Veith FJ, Ohki T, Scher LA, Berdejo GL, Lipsitz EC, et al Histologic and duplex comparison of the perclose and angio-seal percutaneous closure devices. Vascular. 2007; 15: 24–9. 10.2310/6670.2007.00004 17382051

[10] Bangalore S, Arora N, Resnic FS. Vascular Closure Device Failure: Frequency and Implications A Propensity-Matched Analysis. Circ Cardiovasc Interv. 2009; 2: 549–56. 10.1161/CIRCINTERVENTIONS.109.877407 20031773 PMC3046770

[11] Jensen J, Saleh N, Jensen U, Svane B, Jönsson A, Tornvall P. The inflammatory response to femoral arterial closure devices: a randomized comparison among FemoStop, AngioSeal, and Perclose. Cardiovasc Interv Radiol. 2008; 31: 751–5. 10.1007/s00270-008-9323-7 18398639

[12] Park Y, Roh HG, Choo SW, Lee SH, Shin SW, Do YS, et al Prospective Comparison of Collagen Plug (Angio-Seal™) and Suture-Mediated (the Closer S™) Closure Devices at Femoral Access Sites. Kor J Radiol. 2005; 6: 248–55. 10.3348/kjr.2005.6.4.248 16374083 PMC2684972

[13] Steinkamp HJ, Werk M, Beck A, Teichgräber U, Haufe M, Felix R. Excimer laser-assisted recanalisation of femoral arterial stenosis or occlusion caused by the use of Angio-Seal. Eur Radiol. 2001; 11: 1364–70. 10.1007/s003300000793 11519544

[14] Medical SJ. Instruction for use Angio-Seal VIP Vascular Closure Device [Accessed 29 Dec 2015] Available: https://www.google.de/url?sa=t&rct=j&q=&esrc=s&source=web&cd=1&ved=0ahUKEwj5neWN3oDKAhUHXBQKHSCEDVYQFggcMAA&url=https%3A%2F%2Fmanuals.sjm.com%2F~%2Fmedia%2Fmanuals%2Fproduct-manual-pdfs%2F8%2Fd%2F8d810476-6b64-4a38-9fd1-db825e378709.ashx&usg=AFQjCNEDrTPUs8h2j_fulx02Ds0eyjCbAA&bvm=bv.110151844,d.d24.

[15] Applegate RJ, Rankin KM, Little WC, Kahl FR, Kutcher MA. Restick following initial Angioseal use. Cath Cardiovasc Interv. 2003; 58: 181–4. 10.1002/ccd.10419 12552540

[16] Medical SJ. Angio-Seal VIP Vascular Closure Device Brochure. St. Jude Medical [Accessed 29 Dec 2015]. Available: http://professional.sjm.com/products/vas/hemostasis-management/vascular-closure-devices/angio-seal-vip

[17] Abando A, Hood D, Weaver F, Katz S. The use of the Angioseal device for femoral artery closure. J Vasc Surg. 2004; 40: 287–90. 10.1016/j.jvs.2004.05.007 15297822

[18] Silber S. Hemostasis success rates and local complications with collagen after femoral access for cardiac catheterization: analysis of 6007 published patients. Am Heart J. 1998; 135: 152–6. 9453535 10.1016/s0002-8703(98)70356-4

[19] Hoffer EK, Bloch RD. Percutaneous arterial closure devices. J Vasc Interv Radiol 2003; 14: 865–85. 12847195 10.1097/01.rvi.0000071086.76348.8e

[20] Tellez A, Cheng Y, Yi GH, Conditt GB, McGregor JC, Flynn AM, et al In vivo intravascular ultrasound analysis of the absorption rate of the Angio-Seal vascular closure device in the porcine femoral artery. EuroIntervention. 2010; 5: 731–6. 20142226 10.4244/eijv5i6a120

[21] Isfort P, Tanaka T, Penzkofer T, Bruners P, Tolba R, Kuhl CK, et al Vascular closure devices after endovascular procedures in swine: a reliable method? Sci World J. 2014; 2014 10.1155/2014/514942 24737976 PMC3967389

[22] Hofmann LV, Sood S, Liddell RP, Gupta A, Arepally A, Rodriguez ER, et al Arteriographic and pathologic evaluation of two suture-mediated arterial closure devices in a porcine model. J Vasc Interv Radiol. 2003; 14: 755–61. 12817043 10.1097/01.rvi.0000079985.80153.17

[23] Conn PM. Sourcebook of models for biomedical research. 1st ed Totowa: Springer Science & Business Media, 2007.

[24] Hiebl B, Müller C, Hünigen H, Gemeinhardt O, Plendl J, Jung F, et al Gross anatomical variants of the vasculature of the Göttingen TM minipig. Appl Cardiopulm Pathophysiol. 2010; 14: 236–43.

[25] Van Der Giessen WJ, Lincoff AM, Schwartz RS, Van Beusekom HMM, Serruys PW, Holmes DR, et al Marked inflammatory sequelae to implantation of biodegradable and nonbiodegradable polymers in porcine coronary arteries. Circulation. 1996; 94: 1690–7. 8840862 10.1161/01.cir.94.7.1690

[26] Yuki I, Lee D, Murayama Y, Chiang A, Vinters HV, Nishimura I, et al Thrombus organization and healing in an experimental aneurysm model. Part II. The effect of various types of bioactive bioabsorbable polymeric coils. *J Neurosurg*. 2007; 107: 109–120. 10.3171/JNS-07/07/0109 17639880

