# Supplementary figures and images for: Long Term Outcome after Application of the Angio-Seal Vascular Closure Device in Minipigs
Source: PLoS One. 2016 Sep 28;11(9):e0163878. doi: 10.1371/journal.pone.0163878 (PMC5040263; doi:10.1371/journal.pone.0163878)

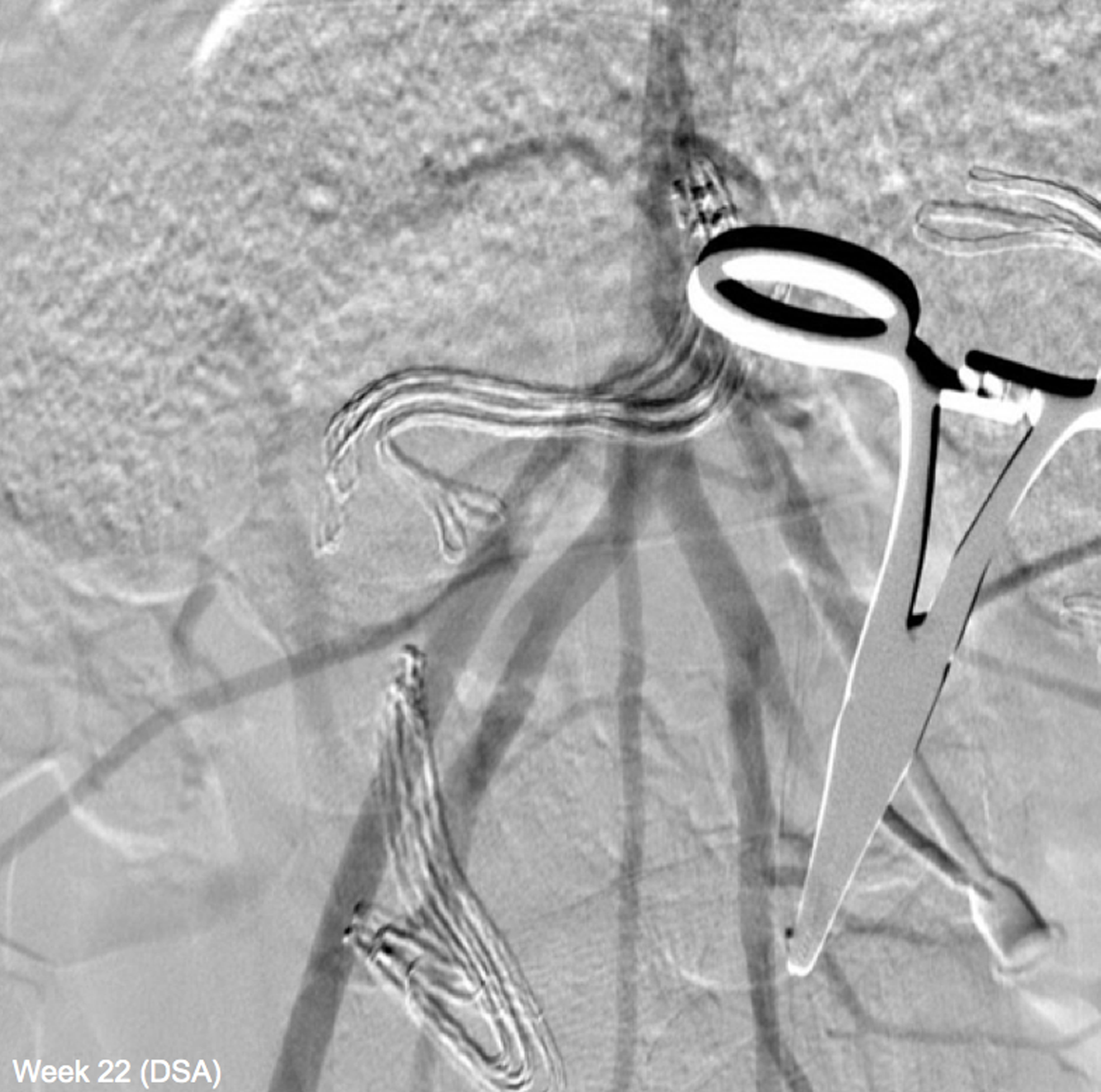

Supplement: S1 Fig — (TIF) [file pone.0163878.s001.tif]

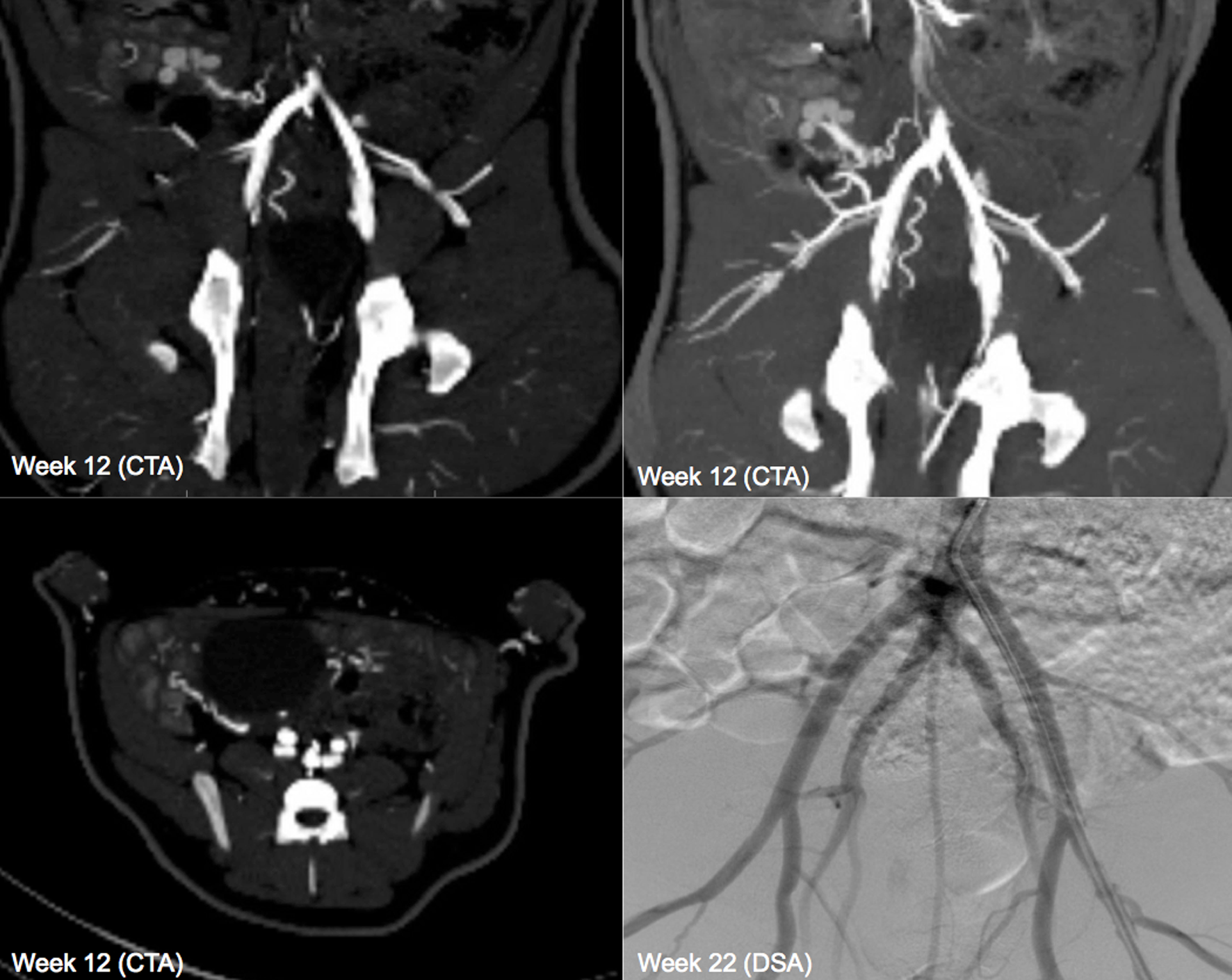

Supplement: S2 Fig — (TIF) [file pone.0163878.s002.tif]

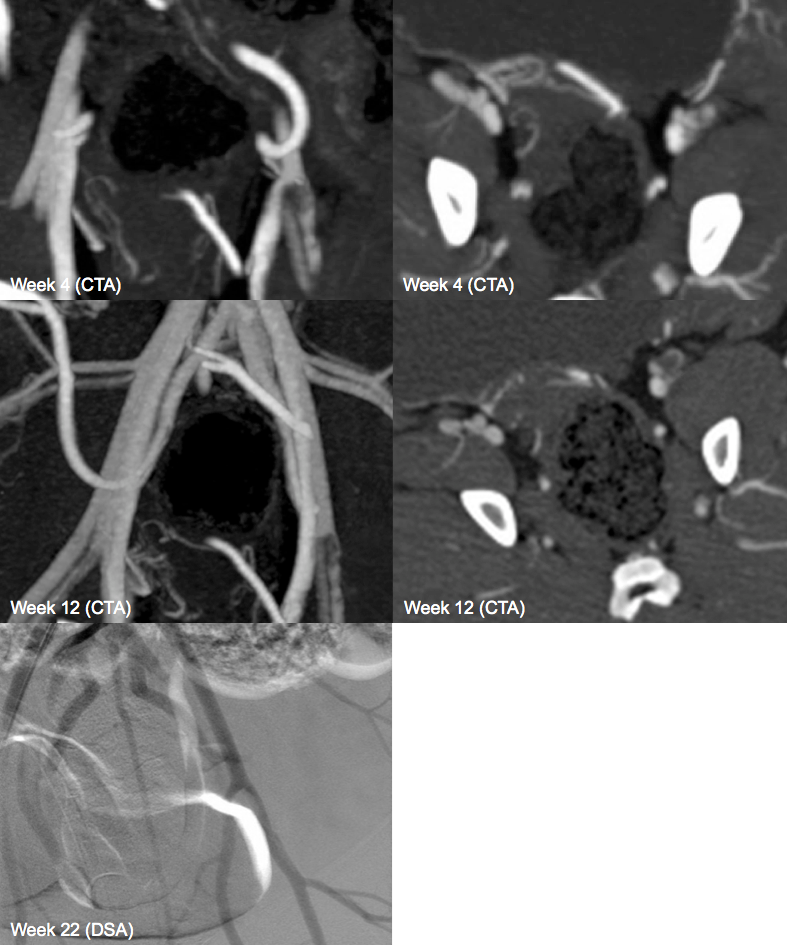

Supplement: S3 Fig — (TIF) [file pone.0163878.s003.tif]

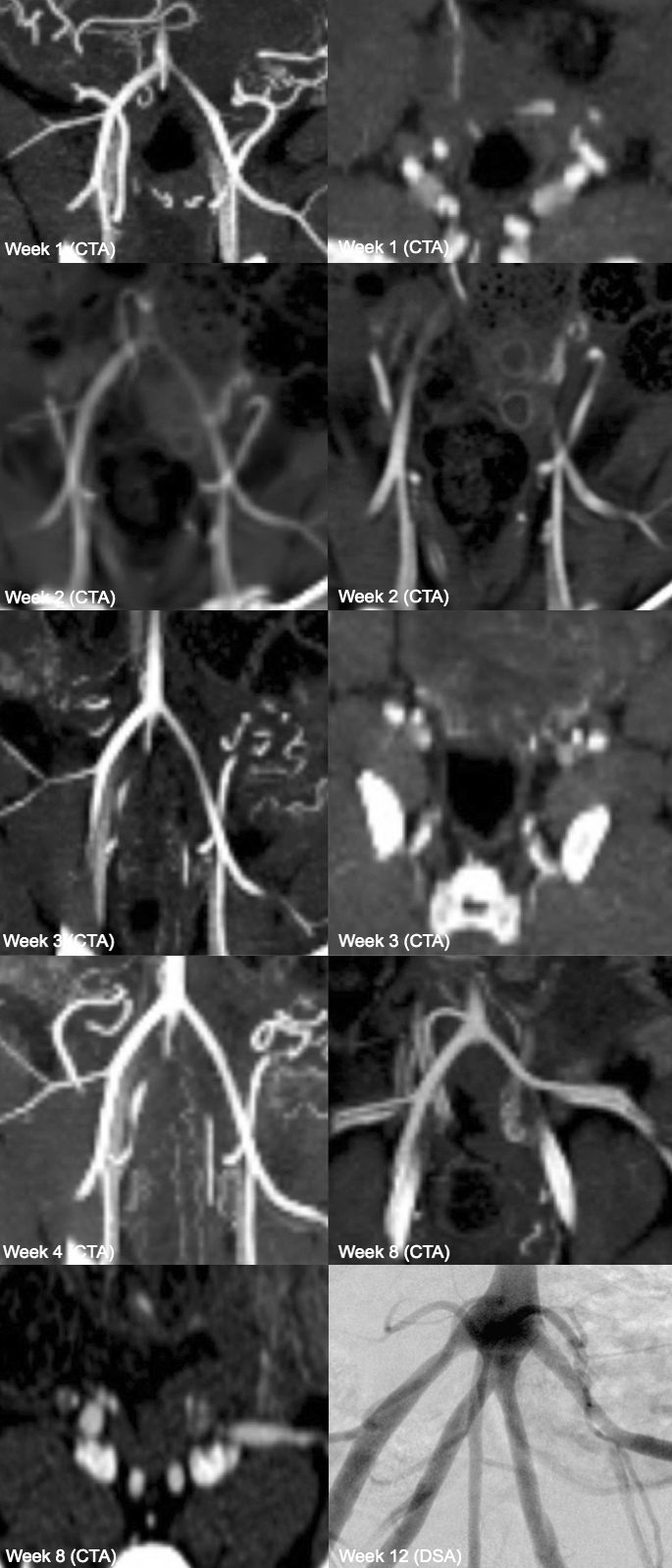

Supplement: S4 Fig — (TIF) [file pone.0163878.s004.tif]

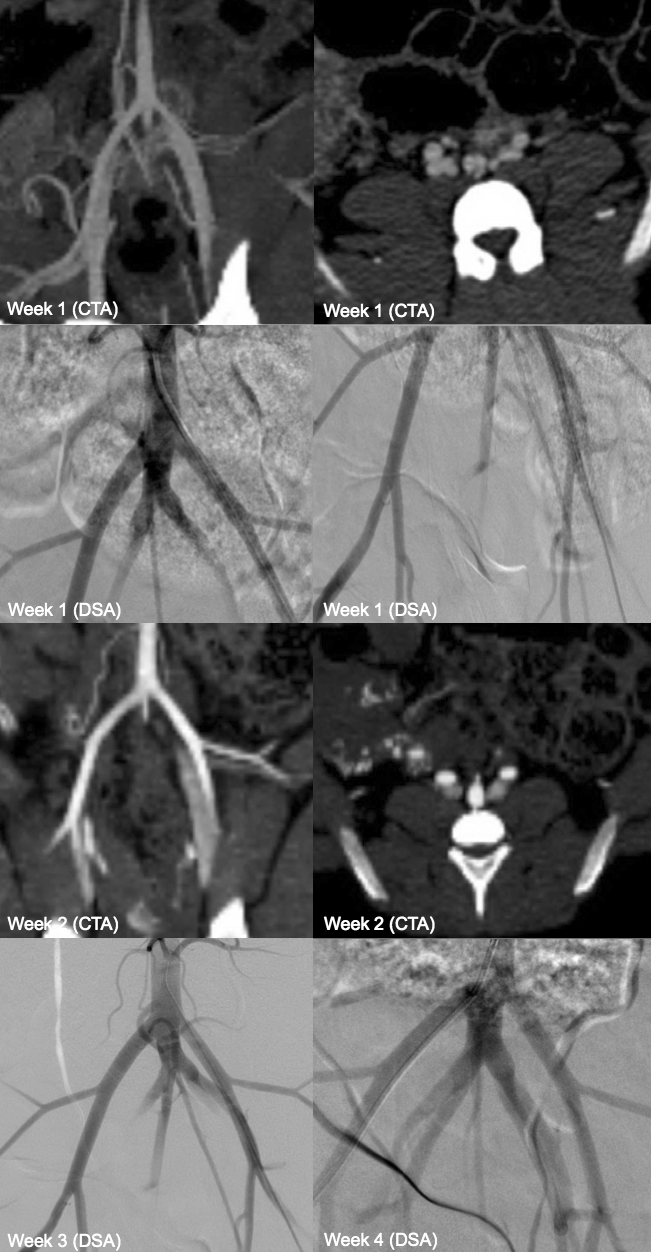

Supplement: S5 Fig — (TIF) [file pone.0163878.s005.tif]

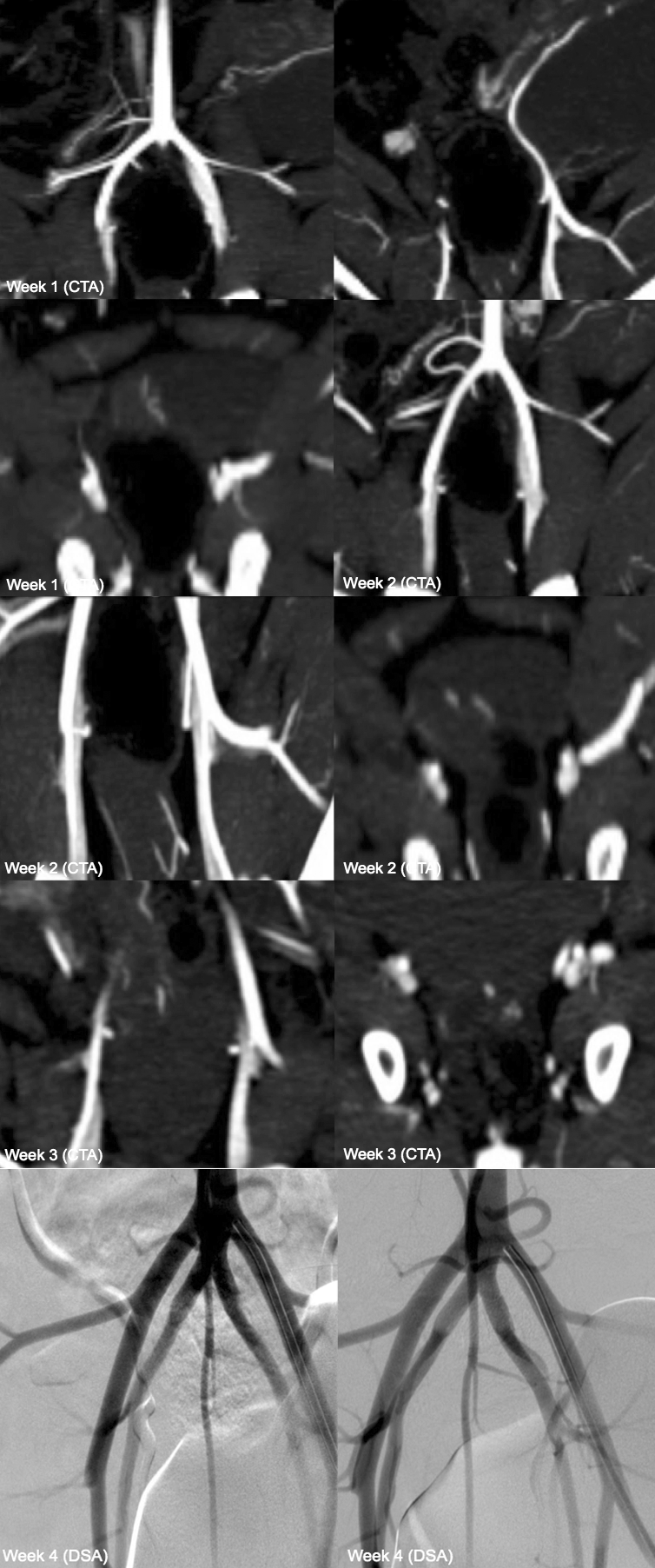

Supplement: S6 Fig — (TIF) [file pone.0163878.s006.tif]

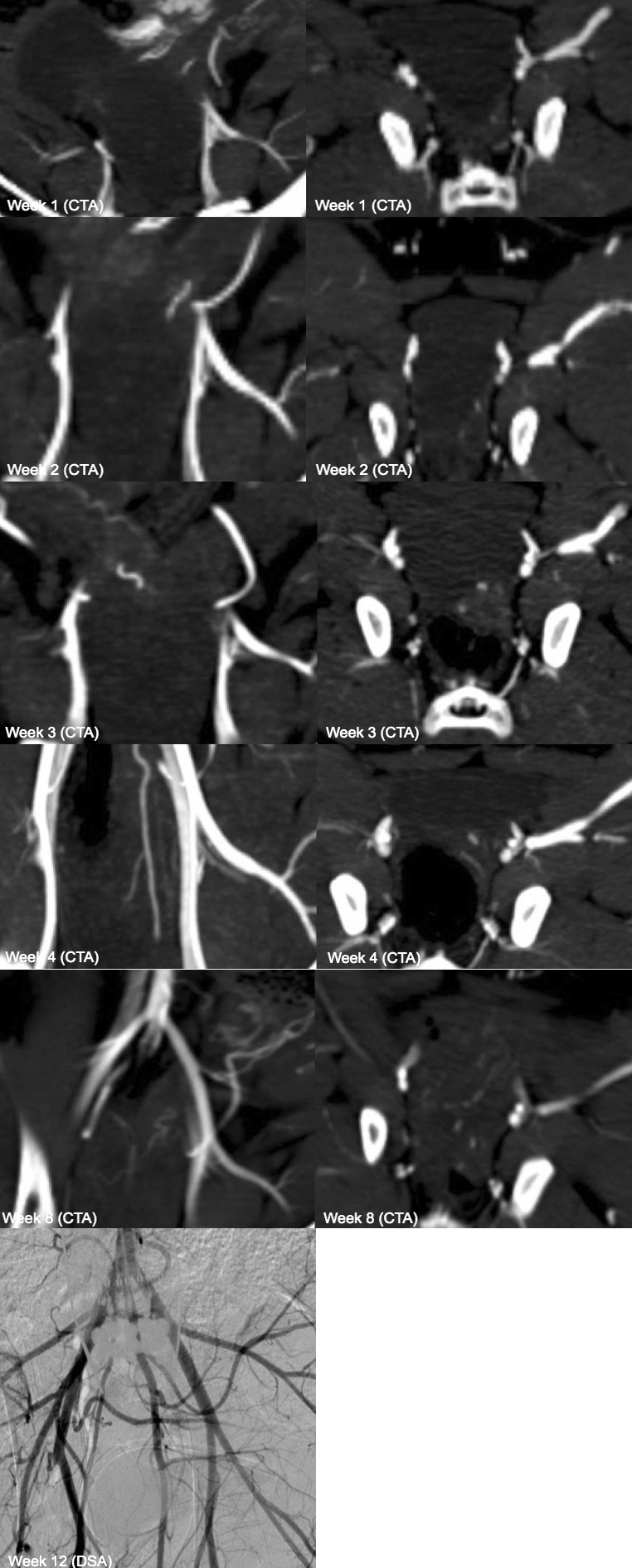

Supplement: S7 Fig — (TIF) [file pone.0163878.s007.tif]

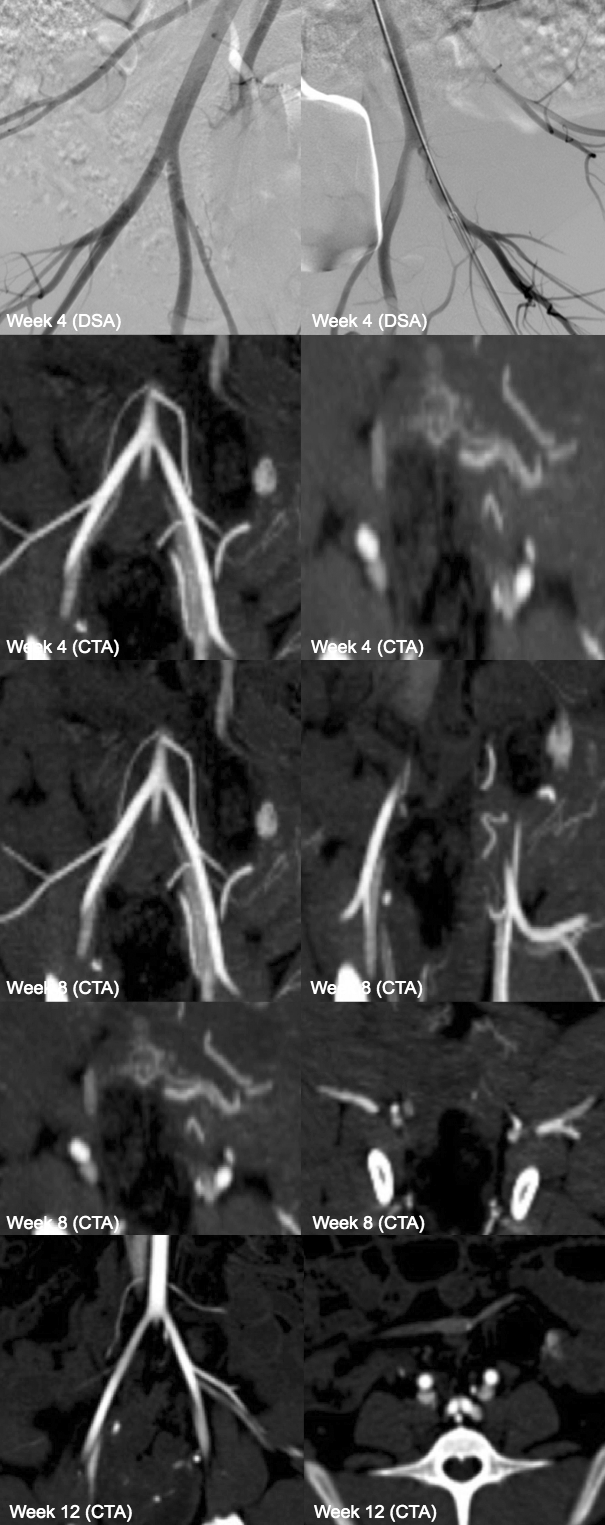

Supplement: S8 Fig — (TIF) [file pone.0163878.s008.tif]
